# Addition of Macrolide Antibiotics for Hospital Treatment of Community-Acquired Pneumonia

**DOI:** 10.1093/infdis/jiae639

**Published:** 2024-12-24

**Authors:** Jia Wei, A Sarah Walker, David W Eyre

**Affiliations:** Nuffield Department of Medicine; Nuffield Department of Medicine; National Institute for Health and Care Research Oxford Biomedical Research Centre; National Institute for Health and Care Research Health Protection Research Unit in Healthcare-Associated Infections and Antimicrobial Resistance; National Institute for Health and Care Research Oxford Biomedical Research Centre; National Institute for Health and Care Research Health Protection Research Unit in Healthcare-Associated Infections and Antimicrobial Resistance; Big Data Institute, Nuffield Department of Population Health, University of Oxford; Department of Infectious Diseases and Microbiology, Oxford University Hospitals NHS Foundation Trust, John Radcliffe Hospital, Oxford, United Kingdom

**Keywords:** community-acquired pneumonia, β-lactam, macrolide, mortality, antimicrobial resistance

## Abstract

**Background:**

Guidelines recommend combining macrolides with β-lactam antibiotics for moderate-to-high severity community-acquired pneumonia (CAP); however, macrolides pose risks of adverse events and anti-microbial resistance.

**Methods:**

We analyzed electronic health data from 8872 adults hospitalized with CAP in Oxfordshire, UK (2016-2024), initially treated with amoxicillin or co-amoxiclav. Using inverse probability treatment weighting, we examined the effects of adjunctive macrolides on 30-day all-cause mortality, time to hospital discharge, and changes in Sequential Organ Failure Assessment (SOFA) score.

**Results:**

There was no evidence of an association between adjunctive macrolides and 30-day mortality (marginal odds ratio 1.05 [95% CI 0.75-1.47] for amoxicillin with vs. without macrolide; 1.12 [0.93-1.34] for co-amoxiclav with vs. without macrolide); and no evidence of a difference in time to discharge (restricted mean days lost +1.76 days [−1.66, +5.19] for amoxicillin, +0.44 days [−1.63, +2.51] for co-amoxiclav). Macrolide use was not associated with SOFA score decreases. Results were consistent across severity sub-groups and sensitivity analyses with missing covariates imputed.

**Conclusions:**

At a population level, the addition of macrolides was not associated with improved clinical outcomes for patients with CAP. The potential benefits of additional macrolides should be weighed against the risks of adverse effects and anti-microbial resistance.

## Teaser

The study found no evidence that combining macrolides with β-lactam antibiotics improved clinical outcomes for patients hospitalized with community-acquired pneumonia, regardless of disease severity. Benefits of additional macrolides should be weighed against risks of adverse effects and resistance.

Antibiotics are the main treatment for bacterial community-acquired pneumonia (CAP). In the United Kingdom (UK), guidelines from the British Thoracic Society and National Institute for Health and Care Excellence recommend amoxicillin for the empirical treatment of low- to moderate-severity pneumonia, and co-amoxiclav for high-severity pneumonia. For moderate- and high-severity pneumonia, these guidelines suggest combining macrolide antibiotics with amoxicillin or co-amoxiclav to provide coverage for atypical pathogens such as *Mycoplasma* [1, 2]. Similarly, European and Latin American guidelines also recommend the addition of macrolides to β-lactams as empirical antibiotic therapy in hospitalized patients with severe CAP [3], while United States (US) guidelines recommend including atypical cover (which can be with a β-lactam + macrolide or respiratory fluroquinolone monotherapy) for all patients hospitalized with CAP [4].

Macrolides have also been hypothesized to improve outcomes from pneumococcal pneumonia, even when an active β-lactam is given, by means other than their antimicrobial activity (eg, anti-inflammatory effects) [5]. However, macrolide use is associated with an increased risk of adverse cardiovascular events [6, 7], adverse gastrointestinal events [8], and resistance against multiple antibiotics at both a population level [9] and within individuals [10].

Existing population-based studies and clinical trials have yielded contradictory evidence.

Several retrospective and small prospective studies have suggested that dual therapy (β-lactam plus macrolide) is associated with lower mortality in patients with severe CAP [11–14]. However, these findings have been challenged by other observational studies that reported no significant differences in outcomes, such as mortality and length of hospital stay, between monotherapy and combination therapy [15–17]. Two meta-analyses of observational studies reported that macrolide use was associated with a significant reduction in mortality [18, 19]. In contrast, a noninferiority trial in non–intensive care unit (ICU) CAP patients found that monotherapy was noninferior to combination therapy in terms of 90-day mortality [20]. Another noninferiority trial recruiting patients with moderately severe CAP reported delayed clinical stability with monotherapy but found no differences in mortality or length of stay [21]. A recent randomized trial suggested that adding macrolides to β-lactam therapy improved clinical response and reduced the inflammatory burden, although mortality remained similar between groups [22].

Given these conflicting results, large observational studies may help by comparing treatment options in real-world representative populations. We therefore examined the effect of adjunctive macrolides on clinical outcomes in adults hospitalized for CAP receiving β-lactam therapy, using electronic healthcare records from a large UK teaching hospital group, extending the approach used in our recent analysis of the impact of β-lactam choice in CAP [23].

## METHODS

### Patients and Setting

De-identified electronic patient record data were obtained from Oxford University Hospitals, UK, using the Infections in Oxfordshire Research Database, which has Research Ethics Committee, Health Research Authority, and Confidentiality Advisory Group approvals (19/SC/0403, 19/CAG/0144) for use of data without individual patient consent.

We included all adults (≥16 years of age) admitted to Oxford University Hospitals between 1 January 2016 and 19 March 2024 with a primary diagnosis code of pneumonia (*International Classification of Diseases, Tenth Revision* [*ICD-10*] J13–J18) in the first episode (or part) of each admission, that is, only considering patients for whom pneumonia was plausibly the reason for hospital admission. We excluded patients with severe acute respiratory syndrome coronavirus 2 (SARS-CoV-2) infection secondary diagnosis codes (*ICD-10* U07.1/U07.2) or admitted from 1 February 2020 to 31 May 2020 (ie, before widespread SARS-CoV-2 testing) to avoid including coronavirus disease 2019 (COVID-19) pneumonia. Linked microbiology and radiology data were used to assess the performance of coding data for identifying pneumonia (Supplementary Methods). Out-of-hospital mortality was available from a national information system recording all UK deaths.

### Exposures, Outcomes, and Covariates

All antibiotics prescribed in the hospital, intravenous/oral and inpatient/postdischarge, received within −12 to +24 hours of admission were considered baseline antibiotics. As main exposures, we compared 4 groups of patients—that is, those who received baseline amoxicillin or co-amoxiclav, with or without an additional macrolide (clarithromycin, azithromycin, or erythromycin). We also included as separate binary variables whether patients received additional doxycycline or gentamicin. Although gentamicin would not provide effective treatment for pneumonia, adding gentamicin to a β-lactam was part of hospital guidelines for empirically managing sepsis with an uncertain source, so provided some adjustment for clinician assessment of disease severity. We excluded patients who received both amoxicillin and co-amoxiclav in the baseline window or who received any antibiotics other than those listed above in the baseline period.

We estimated associations between baseline antibiotics and 30-day all-cause mortality (in-hospital and postdischarge) following admission, time to hospital discharge within the current admission, and change in Sequential Organ Failure Assessment (SOFA) score at 48 hours versus admission. Fifty (0.6%) patients were censored before 30 days (last vital status check or last information in dataset <30 days from admission) and were assumed to be alive at 30 days. SOFA score was calculated from partial pressure of oxygen, fraction of inspired oxygen, platelets, Glasgow Coma Scale, bilirubin, mean arterial pressure, and creatinine [24]. We used measurements that were closest to admission time/date and 48 hours later for SOFA score calculation. Missing components were imputed as scoring 0. For those discharged before 48 hours, we used the measurements closest to their discharge time/date.

To account for disease severity at presentation and its impact on treatment choice, we adjusted for the following baseline covariates: age, sex, ethnicity, index of multiple deprivation percentile, admission specialty, admission hour of day (12 Am to 8 Am, 8 Am to 11 Am, 11 Am to 3 Pm, 3 Pm to 12 Am [25]), admission day of the week, calendar time, hospital admission in the past year (binary), hospital length of stay in the past year, Charlson comorbidity index score, hospital frailty risk score [26], additional specific comorbidities (recent urinary tract infection, immunosuppression, palliation, autoimmune diseases), admission vital signs, laboratory tests, and pneumonia risk prediction scores (CURB-65 [acronym for Confusion, Urea, Respiratory rate, Blood pressure and age ≥65], Pneumonia Severity Index [PSI/PORT], and SMART-COP [acronym for Systolic blood pressure, Multilobar infiltrates, Albumin, Respiratory rate, Tachycardia, Confusion, Oxygen and pH]). Smoking status was not available. For the SOFA score model, we additionally adjusted for baseline SOFA score (see [23] and Supplementary Methods).

### Statistical Analysis

Hospital guidelines were aligned with national recommendations, but there was sufficient variation in prescribing practice to emulate a target trial, that is, to provide a causal estimate of the effect of initial additional macrolide (treatment) versus no macrolide (control). We used inverse probability of treatment weighting (IPTW) to estimate the average treatment effect in the population [27]. IPTW used sampling weights to create a quasi-randomized synthetic sample, truncating extreme weights to optimize covariate balance targeting standardized mean differences <0.1 [28] (Supplementary Table 1 and Supplementary Figure 1). We divided patients into 4 treatment groups (amoxicillin, amoxicillin + macrolide, co-amoxiclav, co-amoxiclav + macrolide), and weights were estimated from propensity scores calculated using multinomial logistic regression with the 4 treatment groups as the outcome, including all baseline covariates and allowing nonlinear and interaction terms. Effects of continuous variables were modeled using natural cubic splines where nonlinear terms improved the model fit by Bayesian information criterion (BIC) >6 [29] in univariable models for 30-day all-cause mortality. The optimal number of knots (2–5) was chosen based on minimizing the BIC. The same transformation was then used in all outcome and treatment models. Continuous variables were truncated at the 2.5th and 97.5th percentiles to avoid undue influence from outliers. Pairwise interactions between main effects were retained in final models if they improved the model fit by BIC >6 [29].

Following weighting, treatment effects on 30-day mortality were estimated using logistic regression with cluster-robust standard errors, calculating marginal odds ratios (ORs), and treatment effects on decreases in SOFA score using linear regression, calculating the marginal mean difference [30]. Treatment effects on time to discharge were estimated using a cause-specific Cox proportional hazards regression model censoring in-hospital death, calculating the marginal difference in restricted mean days lost (RMDL) up to 30 days following admission [31]. RMDL is defined as the area under the cumulative incidence curve up to a specific time point. Time to discharge was censored at 30 days following admission to reduce the bias from very long hospital stays.

We calculated the pairwise contrast between amoxicillin + macrolide versus amoxicillin, and co-amoxiclav + macrolide versus co-amoxiclav as well as testing for heterogeneity in the effect of adjunctive macrolide between those receiving amoxicillin and co-amoxiclav (reported as an interaction *P* value). In the outcome models, we further adjusted for all covariates included in the weighting models to increase the estimate's precision, reduce bias due to residual imbalance, and make the effect estimate “doubly robust” [32].

Subgroup analyses were performed stratified by baseline CURB-65 pneumonia severity score (mild, 0–1; moderate, 2; severe, 3–5). The primary analyses used complete cases. Sensitivity analyses used multiple imputation with chained equations [33], with IPTW applied within each imputed dataset, and pooled marginal effects calculated across 25 imputed datasets using Rubin's rules [34]. Additional sensitivity analyses were restricted to patients with radiologically confirmed CAP and patients without baseline doxycycline.

All analyses were performed in R version 4.3 software using the following packages: tidyverse (version 1.3.2), survey (version 4.2-1), mice (version 3.16.0), WeightIt (version 0.14.2), MatchThem (version 1.1.0), comorbidity (version 1.0.5), sandwich (version 3.0-1), marginaleffects (version 0.13.0), and cobalt (version 4.5.2).

## RESULTS

Between 1 January 2016 and 19 March 2024, 8872 patients admitted with a primary pneumonia diagnostic code received baseline amoxicillin or co-amoxiclav (within −12 to +24 hours of admission) and were included in the analyses (Table 1). Among 3239 (36.5%) and 5633 (63.5%) admissions receiving baseline amoxicillin or co-amoxiclav, 606 (18.7%) and 1821 (32.3%) received additional macrolide antibiotics, respectively. The median age was 78.5 (interquartile range [IQR], 65.3–87.1) years, and 4621 (52.1%) were male. Other baseline characteristics (Table 1), comorbidities (Supplementary Table 2), and covariates (Supplementary Table 3) showed moderate differences by initial treatment received, as expected given guidelines.

**Table 1.** Baseline Characteristics and Clinical Outcomes by Initial Antibiotics Received

Characteristic	Amoxicillin (n = 2633)	Amoxicillin + Macrolide (n = 606)	Co-amoxiclav (n = 3812)	Co-amoxiclav + Macrolide (n = 1821)	Total (N = 8872)	*P* Value
Age, y						<.001
Median (Q1, Q3)	76.8 (60.7, 86.6)	77.7 (65.8, 86.8)	80.6 (69.6, 88.0)	76.1 (62.0, 86.2)	78.5 (65.3, 87.1)	
Sex						<.001
Female	1357 (51.5%)	289 (47.7%)	1785 (46.8%)	820 (45.0%)	4251 (47.9%)	
Male	1276 (48.5%)	317 (52.3%)	2027 (53.2%)	1001 (55.0%)	4621 (52.1%)	
Ethnicity						.14
White	2513 (95.4%)	589 (97.2%)	3662 (96.1%)	1757 (96.5%)	8521 (96.0%)	
Non-White	120 (4.6%)	17 (2.8%)	150 (3.9%)	64 (3.5%)	351 (4.0%)	
IMD score						.14
Median (Q1, Q3)	9.7 (6.2, 15.7)	9.8 (5.9, 14.8)	9.9 (6.1, 15.5)	10.2 (6.4, 16.0)	9.9 (6.1, 15.7)	
Missing, No.	29	9	34	34	106	
Additional gentamicin						<.001
No	2594 (98.5%)	590 (97.4%)	3313 (86.9%)	1570 (86.2%)	8067 (90.9%)	
Yes	39 (1.5%)	16 (2.6%)	499 (13.1%)	251 (13.8%)	805 (9.1%)	
Additional doxycycline						<.001
No	1630 (61.9%)	584 (96.4%)	3305 (86.7%)	1763 (96.8%)	7282 (82.1%)	
Yes	1003 (38.1%)	22 (3.6%)	507 (13.3%)	58 (3.2%)	1590 (17.9%)	
Consultant specialty						<.001
Acute medicine	1167 (44.3%)	271 (44.7%)	1754 (46.0%)	784 (43.1%)	3976 (44.8%)	
Emergency medicine	242 (9.2%)	14 (2.3%)	150 (3.9%)	46 (2.5%)	452 (5.1%)	
Gerontology	678 (25.8%)	160 (26.4%)	957 (25.1%)	521 (28.6%)	2316 (26.1%)	
Infectious diseases	276 (10.5%)	60 (9.9%)	350 (9.2%)	203 (11.1%)	889 (10.0%)	
Other	270 (10.3%)	101 (16.7%)	601 (15.8%)	267 (14.7%)	1239 (14.0%)	
CCI score						<.001
Median (Q1, Q3)	1.0 (0.0, 2.0)	1.0 (0.0, 2.0)	2.0 (1.0, 3.0)	1.0 (1.0, 2.0)	1.0 (1.0, 2.0)	
Frailty score						<.001
Median (Q1, Q3)	4.5 (0.5, 13.1)	4.0 (0.8, 11.4)	10.3 (3.7, 20.1)	5.7 (1.8, 12.8)	7.1 (1.8, 16.0)	
Admitted to hospital in previous year						<.001
No	1514 (57.5%)	345 (56.9%)	1666 (43.7%)	1036 (56.9%)	4561 (51.4%)	
Yes	1119 (42.5%)	261 (43.1%)	2146 (56.3%)	785 (43.1%)	4311 (48.6%)	
Length of hospital stay in previous year (d)						<.001
Median (Q1, Q3)	2.7 (0.4, 16.7)	3.9 (0.5, 16.8)	7.5 (0.9, 33.5)	3.6 (0.5, 21.2)	4.8 (0.6, 25.8)	
Severity by CURB-65 score						<.001
Mild (0–1)	1348 (56.2%)	232 (42.0%)	1354 (37.8%)	676 (39.9%)	3610 (43.9%)	
Moderate (2)	752 (31.4%)	204 (36.9%)	1331 (37.2%)	561 (33.1%)	2848 (34.6%)	
Severe (3–5)	298 (12.4%)	117 (21.2%)	893 (25.0%)	458 (27.0%)	1766 (21.5%)	
Missing, No.	235	53	234	126	648	
Severity by SMART-COP score						<.001
Low	1093 (48.2%)	174 (33.0%)	1045 (30.5%)	372 (23.2%)	2684 (34.3%)	
Moderate	1042 (45.9%)	284 (53.9%)	1798 (52.4%)	840 (52.3%)	3964 (50.6%)	
High	128 (5.6%)	67 (12.7%)	511 (14.9%)	323 (20.1%)	1029 (13.1%)	
Very high	6 (0.3%)	2 (0.4%)	75 (2.2%)	71 (4.4%)	154 (2.0%)	
Missing, No.	364	79	383	215	1041	
Severity by PSI/PORT score						<.001
Low_II	280 (16.7%)	65 (15.1%)	253 (8.6%)	204 (14.1%)	802 (12.3%)	
Low_III	326 (19.5%)	82 (19.1%)	468 (15.9%)	222 (15.4%)	1098 (16.9%)	
Moderate_IV	767 (45.8%)	192 (44.7%)	1346 (45.7%)	612 (42.4%)	2917 (44.9%)	
High_V	302 (18.0%)	91 (21.2%)	877 (29.8%)	407 (28.2%)	1677 (25.8%)	
Missing, No.	958	176	868	376	2378	
30-d mortality						<.001
No	2431 (92.3%)	538 (88.8%)	3053 (80.1%)	1502 (82.5%)	7524 (84.8%)	
Yes	202 (7.7%)	68 (11.2%)	759 (19.9%)	319 (17.5%)	1348 (15.2%)	
Time to discharge, d						<.001
Median (Q1, Q3)	1.0 (0.2, 3.9)	2.2 (0.9, 5.8)	3.2 (1.1, 8.3)	3.7 (1.7, 7.9)	2.6 (0.8, 6.9)	
SOFA score at admission						<.001
Mean (SD)	1.5 (1.2)	1.8 (1.4)	2.2 (1.8)	2.3 (1.9)	2.0 (1.6)	
Decrease in SOFA score at 48 h						<.001
Mean (SD)	0.1 (1.0)	0.2 (1.3)	0.3 (1.5)	0.4 (1.8)	0.3 (1.5)	

Data are presented as No. (%) unless otherwise indicated.

Abbreviations: CCI, Charlson comorbidity index; CURB-65, acronym for confusion, urea, respiratory rate, blood pressure and age ≥65; IMD, index of multiple deprivation; PSI/PORT, pneumonia severity index; Q1, quartile 1; Q3, quartile 3; SD, standard deviation; SOFA, Sequential Organ Failure Assessment; SMART-COP, acronym for systolic blood pressure, multilobar infiltrates, albumin, respiratory rate, tachycardia, confusion, oxygen and pH.

Among 8872 admissions, 5267 (59.4%) had blood cultures performed; 216 (2.4%) had a positive blood culture with a pneumonia-associated pathogen. A total of 1320 (14.9%) were tested with influenza/respiratory syncytial virus (RSV) polymerase chain reaction (PCR); 64 (0.7%) had influenza and 49 (0.6%) had RSV detected. Three hundred eighty-eight (4.4%) patients (predominantly immunosuppressed) were tested with a multiplex respiratory PCR, and 22 patients (0.2%) had *Mycoplasma* detected. A total of 1019 (11.5%) received a *Legionella* urinary antigen test, with only 2 positive results. In patients with positive blood cultures, the most common pathogen identified was *Streptococcus pneumoniae* (141 admissions [1.6% of all admissions]), followed by *Staphylococcus aureus* (22 [0.2%]), *Klebsiella pneumoniae* (17 [0.2%]), *Pseudomonas aeruginosa* (12 [0.1%]), and *Haemophilus influenzae* (10 [0.1%]). One hundred thirty-five of 141 (95.7%) *S pneumoniae* isolates were susceptible to penicillin, and 2 of 141 (1.4%) were resistant. Penicillin susceptibility results for *S aureus* were not routinely reported for blood culture isolates (historically <5% of isolates were susceptible). Five of 10 (50%) *H influenzae* isolates were ampicillin resistant.

A total of 7729 (87.1%) admissions had ≥1 chest X-ray and/or computed tomography scan during hospital admission, of which 5896 (76.3%) were identified as showing evidence of pneumonia based on text matching (Supplementary Methods). Only 150 (1.7%) patients were admitted to the ICU within 24 hours of admission to hospital, and 2174 (24.5%) patients had a low blood pressure consistent with shock (systolic blood pressure <90 mm Hg or diastolic blood pressure <60 mm Hg).

At baseline (within −12 to +24 hours of admission), 2633 (29.7%) patients received amoxicillin without macrolide, with a median duration of all antibiotic treatment, including switching agents/route, of 5.2 (IQR, 5.0–7.2) days (5.0 [IQR, 4.8–6.1] days of amoxicillin). Six hundred six (6.8%) patients received amoxicillin and macrolide, with a median of 6.4 (IQR, 5.0–8.5) total days of antibiotics (5.4 [IQR, 4.6–7.0] days of amoxicillin; 5.0 [IQR, 3.6–7.0] days of macrolides). A total of 3812 (43.0%) patients received co-amoxiclav without macrolide, with a median of 6.7 (IQR, 5.0–10.2) total days of antibiotics (5.8 [IQR, 4.6–7.9] days of co-amoxiclav). A total of 1820 (20.5%) patients received co-amoxiclav and macrolide, with a median of 6.9 (IQR, 5.5–10.0) total days of antibiotics (6.2 [IQR, 4.7–8.0] days of co-amoxiclav; 5.0 [IQR, 2.4–7.0] days of macrolide). The median time from inpatient admission to first hospital antibiotic delivery was 1.7 (IQR, −0.7 to 3.7) hours for all baseline antibiotics (some antibiotics initiated in the emergency department prior to admission), and was 2.7 (IQR, 1.2–4.5) hours for amoxicillin, 0.8 (IQR, −1.5 to 3.0) hours for co-amoxiclav, and 1.6 (IQR, −0.4 to 4.0) hours for macrolides (Supplementary Figure 2). Among those who received a baseline macrolide, the majority received clarithromycin (n = 2409 [99.2%]: 1906 oral [79.1%], 357 intravenous [14.8%], 146 both oral and intravenous in the baseline period [6.1%]), 16 patients received oral azithromycin, and 1 patient received oral erythromycin. Among 2633 patients receiving amoxicillin without macrolide at baseline, 102 (3.9%) received additional macrolide after baseline, starting a median of 3.8 (IQR, 1.8–8.8) days postadmission. Among 3812 patients receiving co-amoxiclav without macrolide at baseline, 238 (6.2%) received additional macrolide after baseline, starting a median of 3.9 (IQR, 1.9–8.8) days postadmission. At baseline, 1590 (17.9%) patients received additional doxycycline, and 805 (9.1%) received additional gentamicin (80 [0.9%] received both macrolides and doxycycline).

Unadjusted 30-day all-cause mortality was highest in patients receiving baseline co-amoxiclav without a macrolide (19.9% [759/381]), followed by co-amoxiclav with a macrolide (17.5% [319/182]), amoxicillin with a macrolide (11.2% [68/606]), and lowest after baseline amoxicillin without a macrolide (7.7% [202/2633]) (Table 1). The median (IQR) time to discharge was 3.2 (1.1–8.3), 3.7 (1.7–7.9), 2.2 (0.9–5.8), and 1.0 (0.2–3.9) days in the 4 groups, respectively. The mean (standard deviation) decrease in SOFA score at 48 hours was 0.3 (1.5), 0.4 (1.8), 0.2 (1.3), and 0.1 (1.0) (Supplementary Figure 3). These crude variations likely reflect differences in prescribing practices based on disease severity and underlying comorbidities.

**Figure 1.**
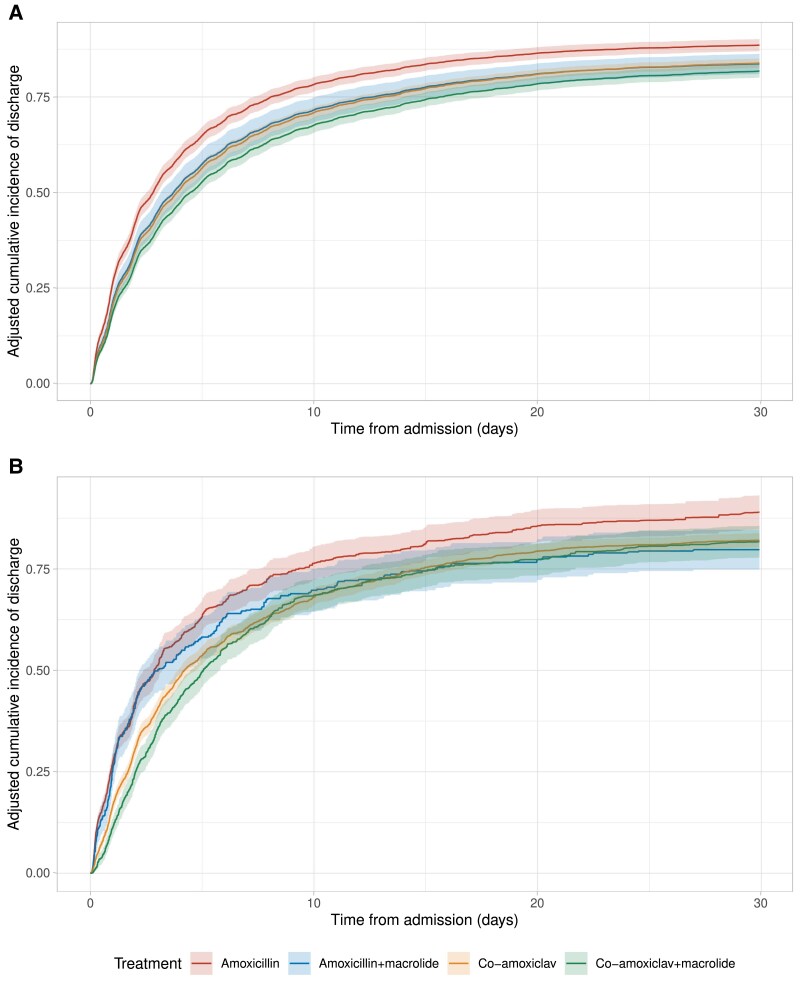
Adjusted cumulative incidence (95% confidence intervals) of hospital discharge by initial antibiotic treatment. Time to discharge was censored at 30 days following admission. *A*, Estimates using multivariable regression without weighting. *B*, Estimates using inverse probability of treatment weighting.

In standard multivariable regression models, macrolides were not associated with 30-day mortality in patients receiving baseline amoxicillin (adjusted odds ratio [OR], 1.10 [95% confidence interval {CI}, .68–1.78]; *P* = .70) or co-amoxiclav (adjusted OR, 1.08 [95% CI, .83–1.39]; *P* = .58), or SOFA score decreases (difference with amoxicillin, −0.01 [95% CI, −.17 to +.15], *P* = .88; co-amoxiclav, +0.03 [95% CI, −.13 to +.06], *P* = .50), but macrolides were associated with longer time to discharge among those receiving baseline amoxicillin (RMDL, 1.72 [95% CI, .70–2.74]; *P* = .001) and co-amoxiclav (RMDL, 0.81 [95% CI, .20–1.43]; *P* = .01) (Table 2, Figure 1*A*).

**Table 2.** Average Treatment Effects of Additional Baseline Macrolide on 30-Day Mortality, Time to Discharge, and Decrease in Sequential Organ Failure Assessment Score

Outcome	Amoxicillin + Macrolide vs Amoxicillin			Co-amoxiclav + Macrolide vs Co-amoxiclav			Interaction
	Marginal OR/Difference	95% CI	*P* Value	Marginal OR/Difference	95% CI	*P* Value	*P* Value
Multivariable regression (without weighting), n = 4893							
30-d mortality	1.10	.68–1.78	.70	1.08	.83–1.39	.58	.92
Time to discharge (RMDL)	+1.72	+.70 to +2.74	.**001**	+0.81	+.20 to +1.43	.**01**	NA^a^
Decrease in SOFA score	−0.01	−.17 to +.15	.88	+0.03	−.13 to +.06	.50	.82
Complete cases (weighting), n = 4893							
30-d mortality	1.05	.75–1.47	.78	1.12	.93–1.34	.24	.62
Time to discharge (RMDL)	+1.76	−1.66 to +5.19	.24	+0.44	−1.63 to +2.51	.68	NA^a^
Decrease in SOFA score	+0.03	−.19 to +.25	.78	−0.06	−.19 to +.06	.29	.16
Multiple imputation (weighting), n = 8872							
30-d mortality	0.92	.65–1.29	.61	1.03	0.89–1.19	.69	.20
Time to discharge (RMDL)	+0.57	−2.78 to +3.92	.74	+0.98	−1.00 to +2.98	.33	NA^a^
Decrease in SOFA score	+0.14	−.09 to +.36	.24	−0.04	−.15 to +.06	.41	.78

Marginal OR is reported for binary outcomes (30-day mortality), and marginal difference is reported for time to event outcome (RMDL, adjusting for the competing risk of in-hospital death) and continuous outcome (decrease in SOFA score). Cumulative incidence of discharge is shown in Supplementary Figure 2. Inverse probability of treatment weighting was used and compared with a standard multivariable regression model. Analyses were performed in complete cases (n = 4893) and the whole dataset with missing measurements imputed (n = 8872). Gray-shaded cells indicate point estimates consistent with benefit from macrolide (see *P* value for evidence of association). *P* values in bold indicate significance (<0.05). Interaction *P* value is reported showing no evidence that the effect of macrolide varied by baseline antibiotic after adjustment.

Abbreviations: CI, confidence interval; NA, not applicable; OR, odds ratio; RMDL, restricted mean days lost; SOFA, Sequential Organ Failure Assessment.

^a^Interaction *P* value is not calculable with cumulative incidence analysis.

After adjustment using IPTWs, there was no evidence of differences in 30-day mortality between patients receiving baseline amoxicillin with versus without macrolides (marginal OR, 1.05 [95% CI, .75–1.47]; *P* = .78) or co-amoxiclav with versus without macrolides (marginal OR, 1.12 [95% CI, .93–1.34]; *P* = .24). There was also no evidence of differences with addition of macrolides in time to discharge among those receiving baseline amoxicillin (RMDL, +1.76 [95% CI, −1.66 to +5.19]; *P* = .24), or co-amoxiclav (RMDL, +0.44 [95% CI, −1.63 to +2.51]; *P* = .68) (Figure 1*B*). There was also no evidence that macrolide use was associated with SOFA score decreases (marginal difference with amoxicillin, +0.03 [95% CI, −.19 to +.25], *P* = .78; co-amoxiclav, −0.06 [95% CI, −.19 to +.06], *P* = .29). There was no evidence that the effects of macrolides varied by baseline β-lactam after adjustment (interaction *P* = .92 for 30-day mortality, *P* = .82 for SOFA score decrease). Results were consistent in sensitivity analyses with missing data imputed (Table 2).

Using CURB-65 scores, 1766 (21.4%) patients had severe, 2848 (34.6%) moderate, and 3610 (43.9%) mild CAP (Supplementary Table 4). Consistent findings were observed for each disease severity group, with no evidence in any group of associations with additional macrolides for 30-day mortality, time to discharge, and SOFA score decreases (Supplementary Table 5 and Supplementary Figure 4). Results were also consistent in sensitivity analyses restricted to 3395 radiologically confirmed CAP patients with complete data, and 3976 patients not receiving baseline doxycycline (Supplementary Table 6).

## DISCUSSION

In this study of 8872 patients hospitalized with CAP, we used causal inference approaches to assess the impact of adding macrolides to β-lactam antibiotics. Our findings showed no significant benefit of adjunctive macrolide therapy on 30-day mortality, time to hospital discharge, or reduction in SOFA score compared to β-lactam monotherapy. These results were consistent across varying levels of pneumonia severity, as assessed by CURB-65 scores, and for both amoxicillin and co-amoxiclav as the baseline treatment.

Mortality is commonly reported as the primary endpoint in CAP studies. Our results showed no evidence of differences in 30-day mortality after weighting, with point estimates of 1.05 for amoxicillin with macrolides versus amoxicillin, and 1.12 for co-amoxiclav with macrolides versus co-amoxiclav, and CIs consistent with at most a 20%–30% benefit, but also at worst up to 30%–40% harm. Several systemic reviews and meta-analyses of retrospective and prospective studies have reported a reduction of 18%–30% in mortality with macrolide use [18, 19, 35]. The discrepancy could be due to unmeasured confounders, different population characteristics, and different circulating pathogens. However, several randomized controlled trials (RCTs) also have not reported differences in 30-day or 90-day mortality between β-lactam monotherapy and β-lactam–macrolide combination therapy [20–22], and a meta-analysis including randomized trials and patients receiving guideline-concordant antibiotics also found no significant differences in mortality [36]. However, the lack of differences identified in some clinical trials could reflect small sample sizes not being adequately powered to detect mortality differences.

We also found no association between macrolide use and time to discharge/length of hospital stay. Length of stay determines both patient experiences and consumption of hospital resources, but is rarely reported in CAP treatment studies. We used a competing risk approach to account for in-hospital death, and compared the confounder-adjusted cause-specific cumulative incidence curves using RMDL. Our results align with a previous retrospective study on CAP patients with *Pseudomonas aeruginosa* [37], and previous RCTs reporting no differences in length of stay between monotherapy and combination therapy with macrolides [20, 21].

We also found no evidence that additional macrolides were associated with larger decreases in SOFA score 48 hours after admission, a measure of the early attenuation of the inflammatory burden. This contradicts a recent RCT, which found that adjunctive clarithromycin reduced respiratory symptom severity score, SOFA score, and procalcitonin levels, recommending combination therapy for patients with severe disease [22]. The discrepancy may, at least in part, be attributed to different underlying populations, namely, CAP patients with a high inflammatory burden in the trial, although we found no evidence to suggest greater benefits from macrolides in severe disease in our analysis (Supplementary Table 5*A*).

Current guidelines recommend the use of macrolides in patients with moderate to severe CAP, to provide coverage of atypical pathogens and for their immunomodulatory effects. However, at a population level, we found no evidence that empirical macrolide combination therapy provided any benefit over β-lactam monotherapy, and atypical pathogens were rarely identified. Furthermore, in subgroup analyses by severity we found no evidence of, or even trend toward, greater potential benefits in the more severe subgroups. Conversely, previous evidence has shown that macrolide use is associated with increased antibiotic resistance and a higher risk of adverse outcomes. For example, previous macrolide use has been associated with macrolide resistance among *S pneumoniae* within individuals and in population-based studies [9, 38]; in children, mass dose azithromycin administration was associated with resistance in *S pneumoniae*, *S aureus*, and *Escherichia coli* [10]. Several large cohort studies reported that azithromycin and clarithromycin were associated with higher risk of adverse cardiovascular events [6, 7, 39, 40], on which basis the US Food and Drug Administration subsequently published warnings on the use of azithromycin [41]. A meta-analysis on 183 RCTs reported an increased rate of gastrointestinal adverse events with macrolide use [8]. Macrolide use can also affect microbiome composition in healthy adults [42]. Given the known risks associated with macrolides, routine empirical use of macrolides should be carefully balanced. Nevertheless, developing better diagnostic tools to accurately identify causative pathogens would be beneficial, allowing clinicians to target antibiotic therapy more effectively and also minimize unnecessary macrolide use.

Limitations of our study include residual confounding from unmeasured/unrecorded factors leading clinicians to prescribe macrolides to more unwell patients, particularly affecting time to discharge analyses, although we adjusted more completely for confounding with our more detailed data than previous studies. We could not adjust for causative pathogens because most patients lacked positive microbiological data, including for atypical infections (identified in <1%). Low rates of organisms requiring macrolides for treatment potentially explain the lack of association, suggesting that better pathogen diagnostics are needed to identify at-risk populations to target additional antibiotics, but still supporting the conclusion that at a population-level empirical macrolide use may have more harms than benefits. However, this may not generalize in settings with different bacterial species or resistance prevalences. Other limitations include using diagnostic codes for CAP identification, which may be imperfect; however, previous studies have shown that diagnostic codes have high positive predictive value for identifying CAP, although they do miss some cases (ie, lower sensitivity) [43, 44]. The large number of admissions included in our analyses precluded individual note review. Further, results remained consistent among patients with radiologically confirmed CAP. We restricted to patients with nonmissing covariates to best control for confounding, but sicker patients may have a higher likelihood of recorded measurements; nevertheless, sensitivity analyses imputing missing values produced broadly similar results. We only examined baseline antibiotics without considering changes over time, reflecting an “intention-to-treat” approach targeting the effect of empiric macrolide prescriptions. We did not account for smoking status, individual clinicians, or prior community antibiotic usage, as they were not available for analysis. Only a very small proportion of patients were admitted to ICU within the baseline period (1.7%), so our conclusions cannot be generalized to this specific patient group. Also, most patients received oral clarithromycin, so conclusions may not generalize to populations prescribed different macrolides. Although macrolide use is associated with individual antimicrobial resistance, risks of resistance at a population level may only be partially influenced by hospital prescribing of macrolides for CAP, as use for other indications and in the community may also play a role. However, in our hospital the main recipients of macrolides were adults (86%), and based on prescriber documented indications, 58% were prescribed for a respiratory infection, whereas only 5% were prescribed for ear, nose, or throat infections, with no clear source recorded in the majority of the remainder.

In conclusion, in hospitalized CAP patients we found no evidence of differences in clinical outcomes associated with adjunctive macrolide antibiotics, regardless of disease severity. Our findings suggest that the benefits of empirical macrolide therapy should be weighed against the risk of resistance and side effects. A sufficiently large-scale multicenter RCT providing estimates with low uncertainty is needed to definitively answer the controversial question of the role of macrolides in CAP.

## Supplementary Material

jiae639_Supplementary_Data

## Supplementary Data

Supplementary materials are available at *The Journal of Infectious Diseases* online (http://jid.oxfordjournals.org/). Supplementary materials consist of data provided by the author that are published to benefit the reader. The posted materials are not copyedited. The contents of all supplementary data are the sole responsibility of the authors. Questions or messages regarding errors should be addressed to the author.

## Notes

***Author contributions.*** All authors designed and planned the study and the analysis. J. W. performed the statistical analysis of the data. All authors drafted the manuscript, contributed to the interpretation of the data and results, revised the manuscript, and approved the final version of the manuscript.

***Acknowledgments.*** This work uses data provided by patients and collected by the UK's National Health Service (NHS) as part of their care and support. We thank all of the people of Oxfordshire who contribute to the Infections in Oxfordshire Research Database. Research Database Team: L. Butcher, H. Boseley, C. Crichton, D. W. Crook, D. Eyre, O. Freeman, J. Gearing (community), R. Harrington, K. Jeffery, M. Landray, A. Pal, T. E. A. Peto, T. P. Quan, J. Robinson (community), J. Sellors, B. Shine, A. S. Walker, and D. Waller. Patient and Public Panel: G. Blower, C. Mancey, P. McLoughlin, and B. Nichols.

***Data sharing.*** The datasets analyzed during the current study are not publicly available, as they contain personal data, but are available from the Infections in Oxfordshire Research Database (https://oxfordbrc.nihr.ac.uk/research-themes/modernising-medical-microbiology-and-big-infectiondiagnostics/iord-about/), subject to an application and research proposal meeting the ethical and governance requirements of the database. For further details on how to apply for access to the data and for a research proposal template, please email iord@ndm.ox.ac.uk.

***Disclaimer.*** The views expressed in this publication are those of the authors and not necessarily those of the NHS, the National Institute for Health and Care Research, the UK Department of Health, or the UK Health Security Agency.

***Financial support***. This study was funded by the National Institute for Health and Care Research (NIHR) Health Protection Research Unit in Healthcare-Associated Infections and Antimicrobial Resistance at Oxford University in partnership with the UK Health Security Agency (NIHR200915) and the NIHR Biomedical Research Centre, Oxford. D. W. E. is supported by a Robertson Fellowship.
