# Supplementary material for: Addition of Macrolide Antibiotics for Hospital Treatment of Community-Acquired Pneumonia
Source: J Infect Dis. 2024 Dec 24;231(4):e713–22. doi: 10.1093/infdis/jiae639 (PMC11998547; doi:10.1093/infdis/jiae639)
Supplement: jiae639_Supplementary_Data [file jiae639_supplementary_data.pdf]

Addition of macrolide antibiotics for hospital treatment of community-acquired pneumonia: Supplementary Methods

Microbiology and radiology

Microbiology test results were linked to CAP admission to identify pneumonia pathogens isolated from blood culture, including *Streptococcus pneumoniae*, *Pseudomonas aeruginosa, Staphylococcus aureus, Mycoplasma pneumoniae*, *Legionella pneumophila*, *Klebsiella pneumoniae, Haemophilus influenzae, Moraxella catarrhalis*, and *Chlamydia pneumoniae.* Positive results for Legionella urinary antigen test, influenza/RSV PCR, and multiplex respiratory PCR were also reported.

Radiology chest X-ray (CXR) reports and chest/pulmonary/thorax/aorta CT scans were also linked to each admission. The presence of pneumonia was considered confirmed if the CXR/CT report contained any of the following keywords using fuzzy string matching: 'pneumonia', 'consolidation', 'infiltrate', 'airspace', 'bronchopneumonia', 'infection', 'infective', 'air bronchogram', 'density', 'pneumonic', 'abscess', 'aspiration', 'cavity', and the keyword was not negated (negation detection was conducted using negspaCy package in Python, which is based on the NegEx algorithm for identifying negated findings in medical records[1]).

Covariates

We calculated and included Charlson comorbidity score and hospital frailty risk score using all inpatient and outpatient diagnosis codes from the year prior to admission and all secondary diagnosis codes from the current admission. We further adjusted for additional specific comorbidities that were potential predictors of mild/severe infection or lower/greater likelihood of mortality, using the same set of diagnostic codes as above:

i. urinary tract infection (UTI): N300, N301, N302, N303, N305, N309, N340, N342, N390, N410, N411, N412, N413, N131, N132, N133, N134, N136, N419, O230, O233, O239, P393, R300, R309;
ii. immunosuppression: B20–24 (AIDS/HIV), 0987, C77-96 (metastatic cancer, haematological malignancies), D80-84 (primary immunodeficiencies), K721, K729, K766, K767 (end-stage liver disease);
iii. palliative care: Z515;

iv. autoimmune diseases[2]: D510, D591, D693, E050, E063, E10, E271, G35, G610, H20, K50, K51, K73, K743, K900, L10, L12, L40, L63, L809, M05, M06, M08, M313, M33, M315, M316, M353, G700, M34, M321, M329, M350, M459.

CURB-65 scores were calculated by summing one point for each of the following: confusion, urea >7mmol/L, respiratory rate ≥30 breaths/min, blood pressure <90mmHg (systolic) or ≤60mmHg (diastolic), age ≥65 years[3]. We included all available baseline vital signs, and considered patients with an AVPU status other than alert or with a Glasgow Coma Scale (GCS) <15 as confused. Patients with any missing measurements of the five components were classified as 'unknown'.

We additionally adjusted for the SMART-COP score[4] and PSI/PORT score[5] for pneumonia severity. The SMART-COP score was calculated by summing scores (shown in parentheses) for the following:

- multi-lobar involvement on chest X-ray (1),
- albumin <35 d/L (1),
- respiratory rate ≥25 breaths/min if age ≤ 50; ≥30 breaths/min if age >50 (1),
- heart rate ≥125 beats/min (1),
- confusion (1),
- PaO_2 <70 mmHg or oxygen saturation ⩽93% or ratio of arterial partial pressure of oxygen to fraction of inspired oxygen (PaO_2/FiO_2) <333 if age ≤50; PaO_2 <60 mmHg or oxygen saturation ⩽90% or PaO_2/FiO_2 <250 if age >50 (2),
- pH<7.35 (2),
- systolic blood pressure <90 mmHg (2).

The PSI/PORT score was calculated by summing scores (shown in parentheses) for the following:

- age (points=age),
- sex (-10 if female, 0 if male),
- nursing home resident (10),
- neoplastic disease (30),
- liver disease history (20),
- congestive heart failure history (10),
- cerebrovascular disease history (10),
- altered mental status (defined as confusion as above, 20),
- respiratory rate ≥30 breaths/min (20),
- systolic blood pressure <90mmHg (20),
- temperature <35°C or >39.9°C (15),
- heart rate ≥125 beats/min (10),
- pH<7.35 (30),

- urea ≥11mmol/L (20),
- sodium <130 mmol/L (20),
- glucose ≥14 mmol/L (10), hematocrit <30% (10),
- partial pressure of oxygen (PaO_2) <8 kPa (10),
- pleural effusion on X-ray (10).

For both scores, components with missing values were assumed to be 0 score in complete case analyses and determined by imputed values in sensitivity analyses.

Admission vital signs were the closest measurements to the date/time of admission obtained in the [-24,+12h] baseline window, to include observations in the Emergency Department before admission. We included the following vital signs (continuous unless otherwise stated):

- heart rate,
- respiratory rate,
- systolic and diastolic blood pressure,
- temperature,
- oxygen saturations,
- use of oxygen (binary),
- litres of oxygen delivered >1 litre/minute (0 if not on oxygen),
- AVPU status (Alert, response to Voice, response to Pain, Unresponsive).

Missing AVPU measurements were completed using the Glasgow Coma Scale (GCS) when available, classified as: 15: Alert, 9-14: Voice, 4-8: Pain, 3: Unresponsive. We assumed patients with missing AVPU and GCS were 'Alert'. National Early Warning Score 2 (NEWS2) values were calculated from vital signs and included in models[6].

Admission laboratory tests were defined similarly, and included

- haemoglobin,
- mean cell volume (MCV),
- neutrophils,
- lymphocytes,
- monocytes,
- eosinophils,
- platelets,
- sodium,
- potassium,
- urea,
- bilirubin,
- alanine transferase (ALT),
- alkaline phosphatase (ALP),

- albumin,
- C-reactive protein (CRP),
- prothrombin time (PT),
- activated partial thromboplastin time (APTT),
- lactate (venous or arterial), pH (dichotomized into <7.25 and ≥7.25 to reflect acidosis).

Creatinine and white cell count were strongly correlated with urea and neutrophils/monocytes respectively (Pearson correlation coefficient>0.8), and hence were excluded from models.

References

1. Chapman, W. W., Bridewell, W., Hanbury, P., Cooper, G. F. & Buchanan, B. G. A simple algorithm for identifying negated findings and diseases in discharge summaries. *J Biomed Inform* **34**, 301–310 (2001).
2. Eaton, W. W. *et al.* The prevalence of 30 ICD-10 autoimmune diseases in Denmark. *Immunol Res* **47**, 228–231 (2010).
3. Lim, W. *et al.* Defining community acquired pneumonia severity on presentation to hospital: an international derivation and validation study. *Thorax* **58**, 377–382 (2003).
4. Charles, P. G. P. *et al.* SMART-COP: a tool for predicting the need for intensive respiratory or vasopressor support in community-acquired pneumonia. *Clin Infect Dis* **47**, 375–384 (2008).
5. Fine, M. J. *et al.* A prediction rule to identify low-risk patients with community-acquired pneumonia. *N Engl J Med* **336**, 243–250 (1997).
6. Williams, B. The National Early Warning Score: from concept to NHS implementation. *Clinical Medicine* **22**, 499–505 (2022).

Covariate	SMD	Balance
**Age**	0.054	Balanced
**Sex-Male**	0.051	Balanced
**Ethnicity-Non-white**	0.007	Balanced
**IMD**	0.096	Balanced
**Additional gentamicin**	0.045	Balanced
**Additional doxycycline**	0.025	Balanced
**Consultant-Acute medicine**	0.019	Balanced
**Consultant-Emergency Medicine**	0.015	Balanced
**Consultant-Gerontology**	0.023	Balanced
**Consultant-Infectious disease**	0.015	Balanced
**Consultant-Other**	0.011	Balanced
**Charlson score1**	0.109	Not Balanced
**Charlson score2**	0.055	Balanced
**Frailty score1**	0.036	Balanced
**Frailty score2**	0.149	Not Balanced
**Frailty score3**	0.094	Balanced
**Previous length of stay1**	0.059	Balanced
**CURB1**	0.150	Not Balanced
**CURB2**	0.066	Balanced
**SMART1**	0.061	Balanced
**SMART2**	0.109	Not Balanced
**PSI1**	0.081	Balanced
**PSI2**	0.073	Balanced
**PSI3**	0.093	Balanced
**Admissions in previous year**	0.051	Balanced
**Palliative care**	0.031	Balanced
**UTI**	0.009	Balanced
**Immunosuppression**	0.028	Balanced
**Autoimmune diseases**	0.016	Balanced
**Admission Date**	0.123	Not Balanced
**Admission hour:0-8**	0.038	Balanced
**Admission hour:8-11**	0.037	Balanced
**Admission hour:11-15**	0.017	Balanced
**Admission hour:15-24**	0.042	Balanced
**Admission: Wednesday**	0.015	Balanced
**Admission: Monday**	0.027	Balanced
**Admission: Tuesday**	0.035	Balanced
**Admission: Thursday**	0.015	Balanced
**Admission: Friday**	0.024	Balanced
**Admission: Saturday**	0.046	Balanced
**Admission: Sunday**	0.021	Balanced
**Heart rate1**	0.067	Balanced
**Heart rate2**	0.067	Balanced
**Respiratory rate**	0.048	Balanced
**SBP1**	0.080	Balanced
**SBP2**	0.069	Balanced
**SBP3**	0.033	Balanced
**DBP1**	0.047	Balanced
**DBP2**	0.039	Balanced
**Temperature1**	0.090	Balanced
**Temperature2**	0.123	Not Balanced
**Saturation1**	0.041	Balanced
**Saturation2**	0.045	Balanced

**Use of oxygen supplement**	0.089	Balanced
**Oxygen flow rate**	0.148	Not Balanced
**AVPU:Alert**	0.055	Balanced
**AVPU:Pain**	0.012	Balanced
**AVPU:Unresponsive**	0.002	Balanced
**AVPU:Voice**	0.039	Balanced
**NEWS2 score**	0.125	Not Balanced
**Haemoglobin**	0.067	Balanced
**MCV1**	0.081	Balanced
**MCV2**	0.120	Not Balanced
**Monocytes1**	0.108	Not Balanced
**Monocytes2**	0.112	Not Balanced
**Neutrophils**	0.073	Balanced
**Lymphocytes1**	0.133	Not Balanced
**Lymphocytes2**	0.037	Balanced
**Eosinophils1**	0.023	Balanced
**Eosinophils2**	0.107	Not Balanced
**Eosinophils3**	0.097	Balanced
**Platelets1**	0.055	Balanced
**Platelets2**	0.073	Balanced
**Platelets3**	0.050	Balanced
**Sodium1**	0.067	Balanced
**Sodium2**	0.064	Balanced
**Sodium3**	0.073	Balanced
**Potassium1**	0.065	Balanced
**Potassium2**	0.040	Balanced
**Potassium3**	0.085	Balanced
**Urea1**	0.083	Balanced
**Urea2**	0.033	Balanced
**Bilirubin**	0.057	Balanced
**ALT**	0.060	Balanced
**ALP**	0.079	Balanced
**Albumin**	0.048	Balanced
**CRP1**	0.060	Balanced
**CRP2**	0.006	Balanced
**CRP3**	0.089	Balanced
**PT1**	0.131	Not Balanced
**PT2**	0.062	Balanced
**PT3**	0.049	Balanced
**PT4**	0.048	Balanced
**APTT**	0.042	Balanced
**Lactate**	0.070	Balanced
**pH<7.25**	0.029	Balanced

Supplementary Table 1. Standardised mean difference (SMD) of covariates among 4 groups of patients (baseline amoxicillin or co-amoxiclav, with or without an additional macrolide) post-inverse probability treatment weighting (IPTW). SMD<0.1 indicates good covariate balance is achieved. Extreme weights were truncated at 99.9% centile. IMD: index of multiple deprivation; UTI: urinary tract infection; SBP: systolic blood pressure; DBP: diastolic blood pressure; MCV: mean cell volume; ALT: alanine transferase; ALP: alkaline phosphatase; CRP: C-reactive protein; PT: prothrombin time; APTT: activated partial thromboplastin time; CURB: CURB-65 score; SMART: SMART-COP score; PSI: PSI/PORT score. Numbers after variables indicate non-linear spline terms. Covariate balance before and after adjustment is shown in Supplementary Figure 1.

	Amoxicillin (N=2633)	Amoxicillin+macrolide (N=606)	Co-amoxiclav (N=3812)	Co-amoxiclav+macrolide (N=1821)	Total (N=8872)	p value
**Had urinary tract infection in previous year**						< 0.001
**No**	2498 (94.9%)	575 (94.9%)	3402 (89.2%)	1721 (94.5%)	8196 (92.4%)	
**Yes**	135 (5.1%)	31 (5.1%)	410 (10.8%)	100 (5.5%)	676 (7.6%)	
**Had palliative care in previous year**						< 0.001
**No**	2527 (96.0%)	567 (93.6%)	3367 (88.3%)	1680 (92.3%)	8141 (91.8%)	
**Yes**	106 (4.0%)	39 (6.4%)	445 (11.7%)	141 (7.7%)	731 (8.2%)	
**Had immunosuppression in previous year**						< 0.001
**No**	2487 (94.5%)	561 (92.6%)	3396 (89.1%)	1660 (91.2%)	8104 (91.3%)	
**Yes**	146 (5.5%)	45 (7.4%)	416 (10.9%)	161 (8.8%)	768 (8.7%)	
**Had autoimmune diseases in previous year**						0.10
**No**	2322 (88.2%)	540 (89.1%)	3297 (86.5%)	1599 (87.8%)	7758 (87.4%)	
**Yes**	311 (11.8%)	66 (10.9%)	515 (13.5%)	222 (12.2%)	1114 (12.6%)	
**Had myocardial infarction in previous year**						0.04
**No**	2395 (91.0%)	551 (90.9%)	3390 (88.9%)	1643 (90.2%)	7979 (89.9%)	
**Yes**	238 (9.0%)	55 (9.1%)	422 (11.1%)	178 (9.8%)	893 (10.1%)	
**Had congestive heart failure in previous year**						< 0.001
**No**	2182 (82.9%)	504 (83.2%)	2983 (78.3%)	1507 (82.8%)	7176 (80.9%)	
**Yes**	451 (17.1%)	102 (16.8%)	829 (21.7%)	314 (17.2%)	1696 (19.1%)	
**Had peripheral vascular disease in previous year**						< 0.001
**No**	2516 (95.6%)	575 (94.9%)	3475 (91.2%)	1702 (93.5%)	8268 (93.2%)	
**Yes**	117 (4.4%)	31 (5.1%)	337 (8.8%)	119 (6.5%)	604 (6.8%)	
**Had cerebrovascular disease in previous year**						< 0.001
**No**	2447 (92.9%)	576 (95.0%)	3424 (89.8%)	1716 (94.2%)	8163 (92.0%)	
**Yes**	186 (7.1%)	30 (5.0%)	388 (10.2%)	105 (5.8%)	709 (8.0%)	
**Had dementia in previous year**						< 0.001
**No**	2341 (88.9%)	528 (87.1%)	3063 (80.4%)	1586 (87.1%)	7518 (84.7%)	
**Yes**	292 (11.1%)	78 (12.9%)	749 (19.6%)	235 (12.9%)	1354 (15.3%)	
**Had chronic pulmonary disease in previous year**						< 0.001
**No**	1755 (66.7%)	381 (62.9%)	2619 (68.7%)	1158 (63.6%)	5913 (66.6%)	
**Yes**	878 (33.3%)	225 (37.1%)	1193 (31.3%)	663 (36.4%)	2959 (33.4%)	
**Had rheumatoid disease in previous year**						0.85
**No**	2485 (94.4%)	572 (94.4%)	3582 (94.0%)	1720 (94.5%)	8359 (94.2%)	
**Yes**	148 (5.6%)	34 (5.6%)	230 (6.0%)	101 (5.5%)	513 (5.8%)	

**Had peptic ulcer disease in previous year**						0.08
**No**	2614 (99.3%)	601 (99.2%)	3760 (98.6%)	1797 (98.7%)	8772 (98.9%)	
**Yes**	19 (0.7%)	5 (0.8%)	52 (1.4%)	24 (1.3%)	100 (1.1%)	
**Had mild liver disease in previous year**						0.05
**No**	2541 (96.5%)	596 (98.3%)	3668 (96.2%)	1749 (96.0%)	8554 (96.4%)	
**Yes**	92 (3.5%)	10 (1.7%)	144 (3.8%)	72 (4.0%)	318 (3.6%)	
**Had diabetes without complications in previous year**						< 0.001
**No**	2196 (83.4%)	491 (81.0%)	3010 (79.0%)	1466 (80.5%)	7163 (80.7%)	
**Yes**	437 (16.6%)	115 (19.0%)	802 (21.0%)	355 (19.5%)	1709 (19.3%)	
**Had diabetes with complications in previous year**						< 0.001
**No**	2564 (97.4%)	584 (96.4%)	3667 (96.2%)	1785 (98.0%)	8600 (96.9%)	
**Yes**	69 (2.6%)	22 (3.6%)	145 (3.8%)	36 (2.0%)	272 (3.1%)	
**Had hemiplegia or paraplegia in previous year**						< 0.001
**No**	2585 (98.2%)	594 (98.0%)	3679 (96.5%)	1765 (96.9%)	8623 (97.2%)	
**Yes**	48 (1.8%)	12 (2.0%)	133 (3.5%)	56 (3.1%)	249 (2.8%)	
**Had renal disease in previous year**						< 0.001
**No**	2301 (87.4%)	532 (87.8%)	3082 (80.8%)	1607 (88.2%)	7522 (84.8%)	
**Yes**	332 (12.6%)	74 (12.2%)	730 (19.2%)	214 (11.8%)	1350 (15.2%)	
**Had cancer in previous year**						< 0.001
**No**	2367 (89.9%)	532 (87.8%)	3167 (83.1%)	1563 (85.8%)	7629 (86.0%)	
**Yes**	266 (10.1%)	74 (12.2%)	645 (16.9%)	258 (14.2%)	1243 (14.0%)	
**Had moderate or severe liver diseases in previous year**						0.13
**No**	2619 (99.5%)	606 (100.0%)	3783 (99.2%)	1811 (99.5%)	8819 (99.4%)	
**Yes**	14 (0.5%)	0 (0.0%)	29 (0.8%)	10 (0.5%)	53 (0.6%)	
**Had metastatic solid tumour in previous year**						< 0.001
**No**	2538 (96.4%)	576 (95.0%)	3539 (92.8%)	1725 (94.7%)	8378 (94.4%)	
**Yes**	95 (3.6%)	30 (5.0%)	273 (7.2%)	96 (5.3%)	494 (5.6%)	
**Had AIDS/HIV in previous year**						0.51
**No**	2632 (100.0%)	606 (100.0%)	3811 (100.0%)	1819 (99.9%)	8868 (100.0%)	
**Yes**	1 (0.0%)	0 (0.0%)	1 (0.0%)	2 (0.1%)	4 (0.0%)	

Supplementary Table 2. Comorbidities by initial antibiotics received.

	Amoxicillin (N=2633)	Amoxicillin+macrolide (N=606)	Co-amoxiclav (N=3812)	Co-amoxiclav+macrolide (N=1821)	Total (N=8872)	p value
**Heart rate**						< 0.001
**Median (Q1, Q3)**	86.0 (74.0, 98.0)	89.0 (77.0, 102.2)	89.0 (77.0, 102.0)	94.0 (81.0, 108.0)	89.0 (77.0, 102.0)	
**Missing, N**	168	38	146	91	443	
**Respiratory rate**						< 0.001
**Median (Q1, Q3)**	19.0 (18.0, 20.0)	19.0 (18.0, 22.0)	19.0 (18.0, 22.0)	20.0 (18.0, 24.0)	19.0 (18.0, 22.0)	
**Missing, N**	168	38	146	91	443	
**Systolic blood pressure**						< 0.001
**Median (Q1, Q3)**	132.0 (117.0, 148.0)	127.0 (113.0, 143.0)	128.0 (109.0, 145.0)	122.0 (107.0, 140.0)	128.0 (112.0, 145.0)	
**Missing, N**	168	38	146	91	443	
**Diastolic blood pressure**						< 0.001
**Median (Q1, Q3)**	72.0 (63.0, 82.0)	69.0 (58.0, 79.0)	68.0 (59.0, 80.0)	67.0 (56.2, 78.0)	69.0 (59.0, 80.0)	
**Missing, N**	168	38	146	91	443	
**Temperature**						< 0.001
**Median (Q1, Q3)**	36.6 (36.1, 37.1)	36.8 (36.3, 37.4)	36.6 (36.1, 37.2)	36.8 (36.2, 37.6)	36.7 (36.1, 37.3)	
**Missing, N**	168	38	146	91	443	
**Oxygen Saturation**						< 0.001
**Median (Q1, Q3)**	96.0 (94.0, 97.0)	95.0 (94.0, 97.0)	95.0 (94.0, 97.0)	95.0 (93.0, 97.0)	95.0 (94.0, 97.0)	
**Missing, N**	185	41	183	104	513	
**Use of supplemental oxygen**						< 0.001
**No**	1894 (76.8%)	368 (64.8%)	2262 (61.7%)	798 (46.1%)	5322 (63.1%)	
**Yes**	571 (23.2%)	200 (35.2%)	1404 (38.3%)	932 (53.9%)	3107 (36.9%)	
**Missing, N**	168	38	146	91	443	
**Oxygen flow rate**						< 0.001
**Median (Q1, Q3)**	0.0 (0.0, 0.0)	0.0 (0.0, 2.0)	0.0 (0.0, 2.0)	2.0 (0.0, 4.0)	0.0 (0.0, 2.0)	
**Missing, N**	168	38	146	91	443	
**AVPU**						< 0.001
**Alert**	2556 (97.1%)	587 (96.9%)	3445 (90.4%)	1668 (91.6%)	8256 (93.1%)	
**Voice**	76 (2.9%)	19 (3.1%)	319 (8.4%)	135 (7.4%)	549 (6.2%)	
**Pain**	1 (0.0%)	0 (0.0%)	32 (0.8%)	15 (0.8%)	48 (0.5%)	
**Unresponsive**	0 (0.0%)	0 (0.0%)	16 (0.4%)	3 (0.2%)	19 (0.2%)	
**NEWS2 score**						< 0.001
**Median (Q1, Q3)**	2.0 (1.0, 4.0)	4.0 (2.0, 6.0)	4.0 (2.0, 6.0)	5.0 (3.0, 7.0)	4.0 (2.0, 6.0)	
**Missing, N**	185	41	183	104	513	

**Haemoglobin**						< 0.001
**Median (Q1, Q3)**	130.0 (116.0, 141.0)	126.0 (113.0, 139.2)	124.0 (110.0, 137.0)	127.0 (114.0, 140.0)	127.0 (113.0, 139.0)	
**Missing, N**	70	18	96	35	219	
**Mean cell volume**						< 0.001
**Median (Q1, Q3)**	90.4 (86.8, 94.2)	89.8 (86.5, 93.8)	90.9 (86.7, 95.4)	89.9 (86.2, 94.4)	90.5 (86.6, 94.7)	
**Missing, N**	70	18	97	36	221	
**Monocytes**						< 0.001
**Median (Q1, Q3)**	0.8 (0.6, 1.1)	0.9 (0.6, 1.2)	0.8 (0.6, 1.1)	0.9 (0.6, 1.2)	0.8 (0.6, 1.1)	
**Missing, N**	79	21	112	44	256	
**Neutrophils**						< 0.001
**Median (Q1, Q3)**	8.0 (5.7, 10.8)	9.6 (6.8, 13.1)	9.7 (6.7, 13.4)	10.9 (7.8, 15.3)	9.3 (6.5, 13.0)	
**Missing, N**	79	21	112	44	256	
**Lymphocytes**						< 0.001
**Median (Q1, Q3)**	1.2 (0.8, 1.7)	1.0 (0.7, 1.6)	0.9 (0.6, 1.4)	0.9 (0.6, 1.4)	1.0 (0.7, 1.5)	
**Missing, N**	79	21	112	44	256	
**Eosinophils**						< 0.001
**Median (Q1, Q3)**	0.1 (0.0, 0.2)	0.0 (0.0, 0.1)	0.0 (0.0, 0.1)	0.0 (0.0, 0.1)	0.0 (0.0, 0.1)	
**Missing, N**	79	22	112	44	257	
**Platelets**						0.36
**Median (Q1, Q3)**	253.0 (203.0, 319.0)	258.0 (200.0, 328.5)	255.0 (195.0, 328.0)	254.0 (198.0, 330.0)	254.0 (199.0, 326.0)	
**Missing, N**	70	19	98	40	227	
**Haematocrit**						< 0.001
**Median (Q1, Q3)**	0.4 (0.4, 0.4)	0.4 (0.3, 0.4)	0.4 (0.3, 0.4)	0.4 (0.3, 0.4)	0.4 (0.3, 0.4)	
**Missing, N**	70	18	98	36	222	
**Sodium**						< 0.001
**Median (Q1, Q3)**	137.0 (135.0, 139.0)	136.0 (134.0, 139.0)	137.0 (134.0, 139.0)	136.0 (133.0, 139.0)	137.0 (134.0, 139.0)	
**Missing, N**	74	17	93	33	217	
**Potassium**						0.002
**Median (Q1, Q3)**	4.1 (3.8, 4.4)	4.1 (3.7, 4.5)	4.1 (3.8, 4.5)	4.1 (3.7, 4.5)	4.1 (3.8, 4.5)	
**Missing, N**	166	22	192	55	435	
**Urea**						< 0.001
**Median (Q1, Q3)**	6.1 (4.4, 8.8)	6.8 (4.9, 9.9)	7.4 (5.3, 11.4)	7.3 (5.0, 11.1)	6.9 (4.9, 10.5)	
**Missing, N**	76	16	94	35	221	
**Bilirubin**						< 0.001
**Median (Q1, Q3)**	11.0 (7.0, 16.0)	11.0 (8.0, 17.0)	11.0 (8.0, 17.0)	12.0 (8.0, 18.0)	11.0 (8.0, 17.0)	

**Missing, N**	227	48	281	141	697	
**Alanine transaminase**						0.003
**Median (Q1, Q3)**	18.0 (13.0, 29.0)	19.0 (13.0, 29.0)	18.0 (12.0, 30.5)	20.0 (13.0, 31.2)	19.0 (13.0, 30.0)	
**Missing, N**	229	48	281	141	699	
**Alkaline phosphatase**						< 0.001
**Median (Q1, Q3)**	87.0 (70.0, 112.0)	90.0 (71.0, 115.8)	95.0 (74.0, 126.0)	93.0 (73.0, 124.0)	91.0 (72.0, 121.0)	
**Missing, N**	222	44	266	137	669	
**Albumin**						< 0.001
**Median (Q1, Q3)**	34.0 (30.0, 37.0)	31.0 (28.0, 34.0)	31.0 (27.0, 34.0)	30.0 (26.0, 33.5)	31.0 (28.0, 35.0)	
**Missing, N**	214	43	247	134	638	
**C-reactive protein**						< 0.001
**Median (Q1, Q3)**	56.3 (17.9, 125.9)	103.5 (37.7, 188.2)	85.1 (33.0, 166.6)	129.9 (53.5, 250.7)	84.2 (30.0, 171.4)	
**Missing, N**	179	47	233	120	579	
**Prothrombin time**						< 0.001
**Median (Q1, Q3)**	10.9 (10.4, 11.6)	11.4 (10.9, 12.4)	11.4 (10.8, 12.4)	11.6 (11.0, 12.5)	11.3 (10.7, 12.2)	
**Missing, N**	339	122	553	294	1308	
**Activated partial thromboplastin time**						< 0.001
**Median (Q1, Q3)**	25.1 (23.1, 27.7)	25.5 (23.6, 28.3)	25.7 (23.4, 28.9)	26.1 (23.5, 29.6)	25.6 (23.3, 28.6)	
**Missing, N**	347	128	581	307	1363	
**Lactate**						< 0.001
**Median (Q1, Q3)**	1.3 (1.0, 1.8)	1.4 (1.1, 2.0)	1.5 (1.1, 2.2)	1.6 (1.2, 2.3)	1.5 (1.1, 2.1)	
**Missing, N**	836	135	717	280	1968	
**pH<7.25**						< 0.001
**No**	2619 (99.5%)	604 (99.7%)	3717 (97.5%)	1754 (96.3%)	8694 (98.0%)	
**Yes**	14 (0.5%)	2 (0.3%)	95 (2.5%)	67 (3.7%)	178 (2.0%)	

Supplementary Table 3. Covariates (vital signs and laboratory test measurements) by initial antibiotics received.

	Severe CAP (N=1,766)		Moderate CAP (N=2,848)		Mild CAP (N=3,610)	
**30-day mortality**	Alive	Died	Alive	Died	Alive	Died
**Amoxicillin**	253 (85%)	45 (15%)	654 (87%)	98 (13%)	1308 (97%)	40 (3%)
**Amoxicillin+macrolide**	93 (79%)	24 (21%)	168 (82%)	36 (18%)	226 (97%)	6 (3%)
**Co-amoxiclav**	578 (65%)	315 (35%)	1030 (77%)	301 (23%)	1243 (92%)	111 (8%)
**Co-amoxiclav+macrolide**	313 (68%)	145 (32%)	449 (80%)	112 (20%)	626 (93%)	50 (7%)
**Time to discharge (days)**	median	IQR	median	IQR	median	IQR
**Amoxicillin**	2.3	0.8-7.8	2.0	0.7-6.9	0.4	0.2-2.1
**Amoxicillin+macrolide**	3.1	1.3-8.5	2.9	1.0-7.8	1.3	0.5-3.1
**Co-amoxiclav**	4.7	2.0-10.1	3.9	1.7-9.5	2.2	0.8-5.3
**Co-amoxiclav+macrolide**	4.9	2.4-9.9	4.6	2.1-9.1	2.7	1.2-5.2
**Decrease in SOFA score at 48h**	mean	SD	mean	SD	mean	SD
**Amoxicillin**	0.4	1.5	0.2	1.1	0.1	0.8
**Amoxicillin+macrolide**	0.6	1.9	0.1	1.3	0.1	0.8
**Co-amoxiclav**	0.9	2.0	0.1	1.6	0.1	1.0
**Co-amoxiclav+macrolide**	1.0	2.4	0.3	1.8	0.2	1.4

Supplementary Table 4. Clinical outcomes (crude) in patients hospitalised with CAP by baseline antibiotics and disease severity. Severity was determined by baseline CURB-65 pneumonia severity score, classifying scores 0-1 as mild, 2 as moderate, and 3-5 as moderate/severe. IQR: interquartile range. SD: standard deviation.

Outcome	Amoxicillin + macrolide vs Amoxicillin			Co-amoxiclav + macrolide vs Co-amoxiclav			Interaction
	Marginal OR/difference	95%CI	p-value	Marginal OR/difference	95%CI	p-value	p-value
**(A) Severe (N=1,766)**							
**Multivariable regression (Without weighting)**							
**30-day mortality**	1.01	0.39, 2.57	0.99	1.05	0.68, 1.64	0.82	0.93
**Time to discharge (RMDL)**	+3.30	+1.10, +5.49	**0.003**	+0.41	-0.91, +1.73	0.54	NA*
**Decrease in SOFA score**	-0.25	-0.76, +0.26	0.34	+0.06	-0.18, +0.31	0.61	0.25
**Complete cases (weighting)**							
**30-day mortality**	1.27	0.58, 2.76	0.54	1.11	0.78, 1.58	0.57	0.57
**Time to discharge (RMDL)**	+2.17	-13.57, +17.92	0.78	-0.01	-3.68, +3.66	0.99	NA*
**Decrease in SOFA score**	+0.004	-0.63, +0.64	0.99	+0.01	-0.27, +0.29	0.94	0.97
**Multiple imputation (weighting)**							
**30-day mortality**	1.70	0.82, 3.52	0.15	1.08	0.82, 1.45	0.57	**0.02**
**Time to discharge (RMDL)**	+3.79	-20.96, +28.54	0.83	+2.28	-1.58, +6.14	0.25	NA*
**Decrease in SOFA score**	+0.48	-0.28, +1.25	0.22	+0.04	-0.18, +0.28	0.68	0.10
**(B) Moderate (N=2,848)**							
**Multivariable regression (Without weighting)**							
**30-day mortality**	1.03	0.50, 2.14	0.94	1.07	0.70, 1.64	0.74	0.92
**Time to discharge (RMDL)**	+1.08	-0.65, +2.81	0.22	+1.25	+0.15, +2.34	**0.03**	NA*
**Decrease in SOFA score**	+0.06	-0.21, +0.33	0.64	-0.11	-0.28, +0.06	0.22	0.27
**Complete cases (weighting)**							
**30-day mortality**	0.99	0.57, 1.75	0.99	1.01	0.75, 1.36	0.94	0.95
**Time to discharge (RMDL)**	+1.21	-7.02, +9.43	0.77	+1.25	-5.28, +7.80	0.71	NA*
**Decrease in SOFA score**	+0.11	-0.21, +0.43	0.50	-0.15	-0.37, +0.06	0.15	**0.02**
**Multiple imputation (weighting)**							
**30-day mortality**	1.23	0.72, 2.10	0.44	1.06	0.85, 1.38	0.66	0.39
**Time to discharge (RMDL)**	+1.01	-10.10, +12.13	0.86	+0.35	-4.14, +4.84	0.88	NA*
**Decrease in SOFA score**	+0.19	-0.11, +0.50	0.22	-0.12	-0.28, +0.03	0.12	**0.03**

Outcome	Amoxicillin + macrolide vs Amoxicillin			Co-amoxiclav + macrolide vs Co-amoxiclav			Interaction
	Marginal OR/difference	95%CI	p-value	Marginal OR/difference	95%CI	p-value	p-value
**(C) Mild (N=3,610)**							
**Multivariable regression (Without weighting)**							
**30-day mortality**	0.96	0.27, 3.44	0.95	1.08	0.55, 2.14	0.82	0.87
**Time to discharge (RMDL)**	+1.18	-0.04, +2.40	0.06	+0.48	-0.28, +1.24	0.22	NA*
**Decrease in SOFA score**	-0.005	-0.18, +0.17	0.96	-0.001	-0.11, +0.11	0.99	0.97
**Complete cases (weighting)**							
**30-day mortality**	0.99	0.43, 2.31	0.98	1.25	0.68, 2.33	0.45	0.44
**Time to discharge (RMDL)**	-2.06	-19.34, +15.22	0.83	+1.40	-3.91, +6.72	0.61	NA*
**Decrease in SOFA score**	-0.08	-0.29, +0.15	0.55	-0.02	-0.19, +0.14	0.78	0.58
**Multiple imputation (weighting)**							
**30-day mortality**	0.60	0.24, 1.46	0.26	1.25	0.77, 2.03	0.36	**0.002**
**Time to discharge (RMDL)**	-2.84	-12.06, +6.36	0.54	+0.79	-11.62, +13.21	0.90	NA*
**Decrease in SOFA score**	-0.03	-0.20, +0.14	0.71	+0.06	-0.15, +0.10	0.73	**0.04**

Supplementary Table 5. Average treatment effects (marginal odds ratios (ORs)/difference and 95% confidence intervals (CIs)) of additional baseline macrolide on 30-day mortality, length of stay ≥3 days, and decrease in SOFA score in subgroup analyses stratified by baseline pneumonia severity. Marginal odds ratio is reported for binary outcomes (30-day mortality), and marginal difference is reported for time to event outcome (restricted mean days lost (RMDL), adjusted for the competing risk of in-hospital death) and continuous outcome (decrease in SOFA score). Cumulative incidences of discharge before in-hospital death are shown in **Supplementary Figure 2**. Severity was determined by CURB-65 score: severe pneumonia (score 3-5), moderate pneumonia (score 2), and mild pneumonia (score 0-1). Inverse probability treatment weighting (IPTW) was used and compared with a standard multivariable regression model. Analyses were performed in complete cases (N=4,893) and whole dataset with missing measurements imputed (N=8,224, 648 were unknown severity due to missingness in CURB-65 score). Grey cells indicate point estimates consistent with benefit from macrolide (see p-value for evidence of association). Interaction p-value is reported showing the level of evidence that the effect of macrolide varied by baseline antibiotic after adjustment. *Interaction p-value is not calculable with cumulative incidence analysis. SOFA: Sequential Organ Failure Assessment.

Outcome	Amoxicillin + macrolide vs Amoxicillin			Co-amoxiclav + macrolide vs Co-amoxiclav			Interaction
	Marginal OR/difference	95%CI	p-value	Marginal OR/difference	95%CI	p-value	p-value
**(A) Radiologically confirmed (N=3,395)**							
**Multivariable regression (Without weighting)**							
**30-day mortality**	1.34	0.72, 2.49	0.35	1.08	0.78, 1.52	0.62	0.54
**Time to discharge (RMDL)**	+2.21	1.04, +3.34	**0.0003**	+1.10	+0.41, +1.78	**0.001**	NA*
**Decrease in SOFA score**	+0.04	-0.13, +0.22	0.64	-0.05	-0.16, +0.05	0.34	0.35
**Complete cases (weighting)**							
**30-day mortality**	1.14	0.72, 1.84	0.57	1.22	0.92, 1.64	0.16	0.66
**Time to discharge (RMDL)**	+0.97	-4.00, +5.93	0.70	-1.07	-4.81, +2.66	0.57	NA*
**Decrease in SOFA score**	+0.02	-0.21, +0.25	0.85	-0.08	-0.21, +0.05	0.21	0.16
**(B) Without doxycycline (N=3,976)**							
**Multivariable regression (Without weighting)**							
**30-day mortality**	1.23	0.74, 2.08	0.42	1.05	0.81, 1.36	0.69	0.58
**Time to discharge (RMDL)**	+2.27	+1.18, +3.36	**<0.0001**	+0.89	+0.24, +1.54	**0.008**	NA*
**Decrease in SOFA score**	+0.02	-0.16, +0.20	0.83	-0.03	-0.14, +0.07	0.52	0.61
**Complete cases (weighting)**							
**30-day mortality**	1.17	0.78, 1.76	0.45	1.10	0.91, 1.32	0.32	0.65
**Time to discharge (RMDL)**	+2.20	-2.98, +7.40	0.30	+0.87	-0.20, +1.95	0.12	NA*
**Decrease in SOFA score**	+0.07	-0.17, +0.32	0.55	-0.09	-0.21, +0.03	0.13	0.05

Supplementary Table 6. Average treatment effects (marginal odds ratios (ORs)/difference and 95% confidence intervals (CIs)) of additional baseline macrolide on 30-day mortality, time to discharge, and decrease in SOFA score in sensitivity analyses. (A) Among patients with radiologically confirmed pneumonia (N=3,395). **(B)** Among patients without initial doxycycline (N=3,976). Marginal odds ratio is reported for binary outcomes (30-day mortality), and marginal difference is reported for time to event outcome (restricted mean days lost (RMDL) adjusted for in-hospital death as a competing risk) and continuous outcome (decrease in SOFA score). Inverse probability treatment weighting (IPTW) was used and compared with a standard multivariable regression model. Grey cells indicate point estimates consistent with benefit from

macrolide (see p-value for evidence of association). Interaction p-value is reported showing the level of evidence that the effect of macrolide varied by baseline antibiotic after adjustment. *Interaction p-value is not calculable with cumulative incidence analysis. SOFA: Sequential Organ Failure Assessment.

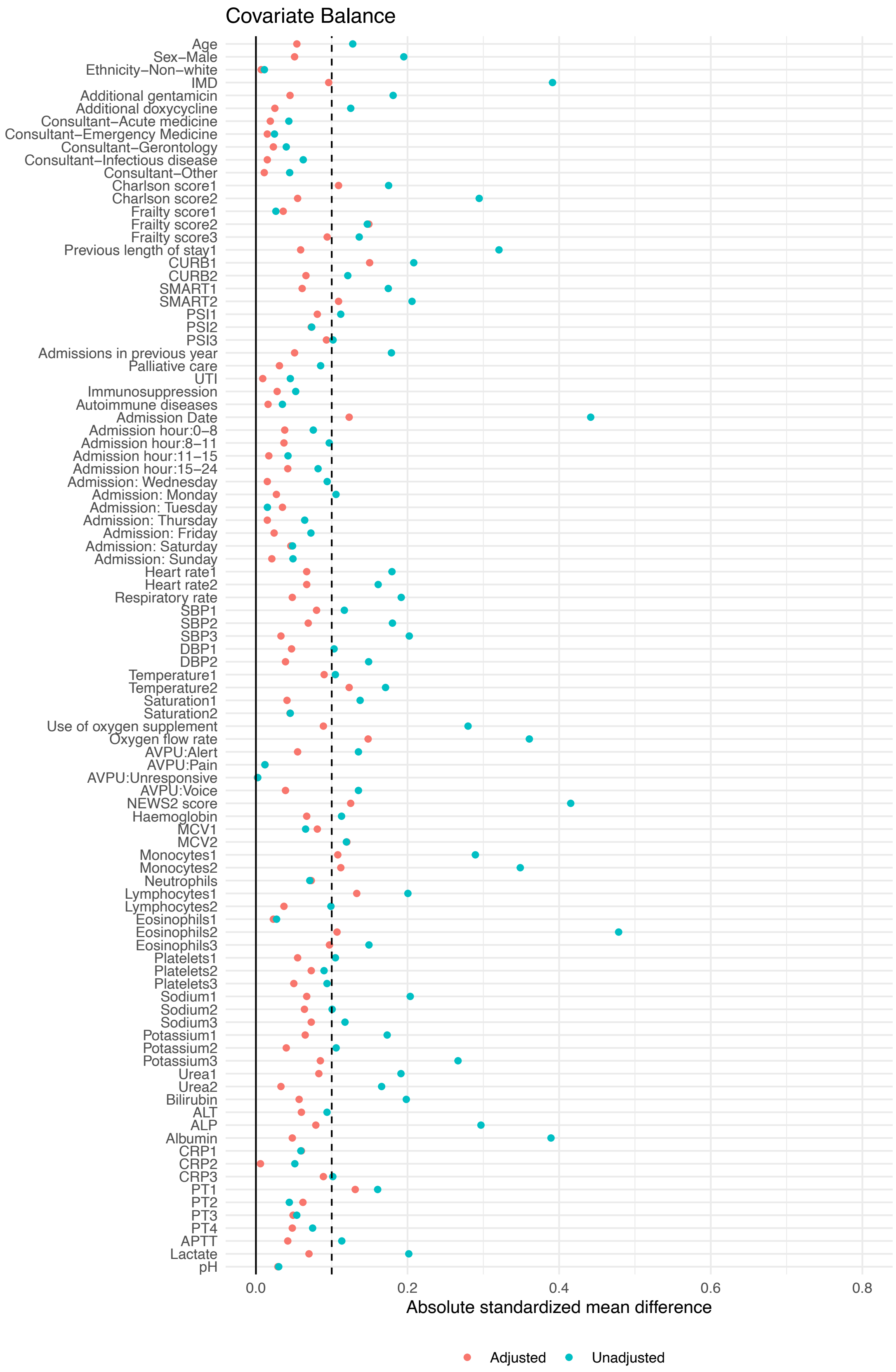

Supplementary Figure 1. Covariate balance before and after inverse probability treatment weighting (IPTW). Dashed line indicates the balance threshold (standardised mean difference=0.1). IMD: index of multiple deprivation; UTI: urinary tract infection; SBP: systolic blood pressure; DBP:

diastolic blood pressure; MCV: mean cell volume; ALT: alanine transferase; ALP: alkaline phosphatase; CRP: C-reactive protein; PT: prothrombin time; APTT: activated partial thromboplastin time; CURB: CURB-65 score; SMART: SMART-COP score; PSI: PSI/PORT score. Numbers after variables indicate non-linear spline terms.

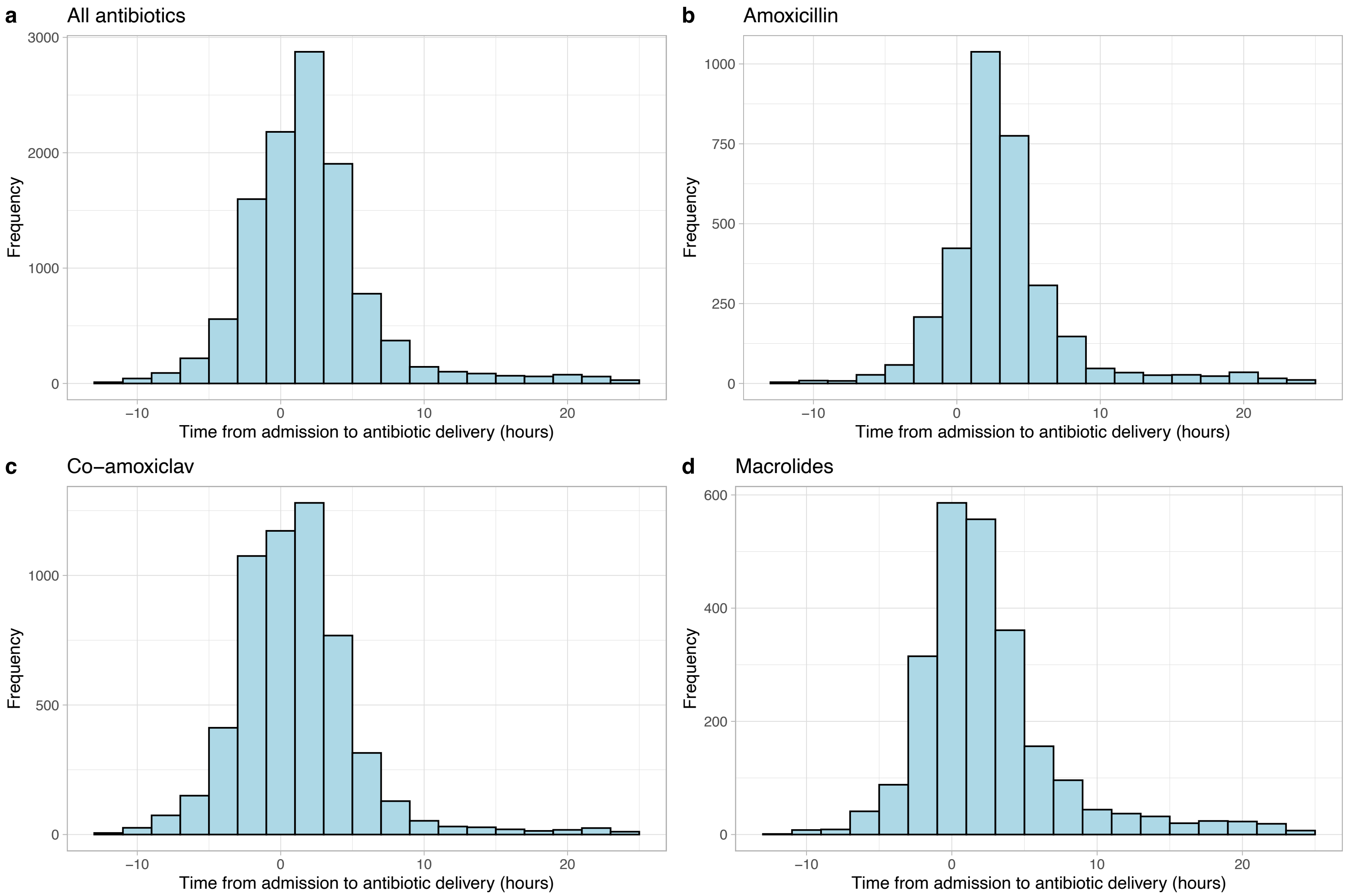

Supplementary Figure 2. Time from admission to antibiotic delivery in hours. a) All baseline antibiotics. b) Baseline amoxicillin. c) Baseline co-amoxiclav. d) Baseline macrolides.

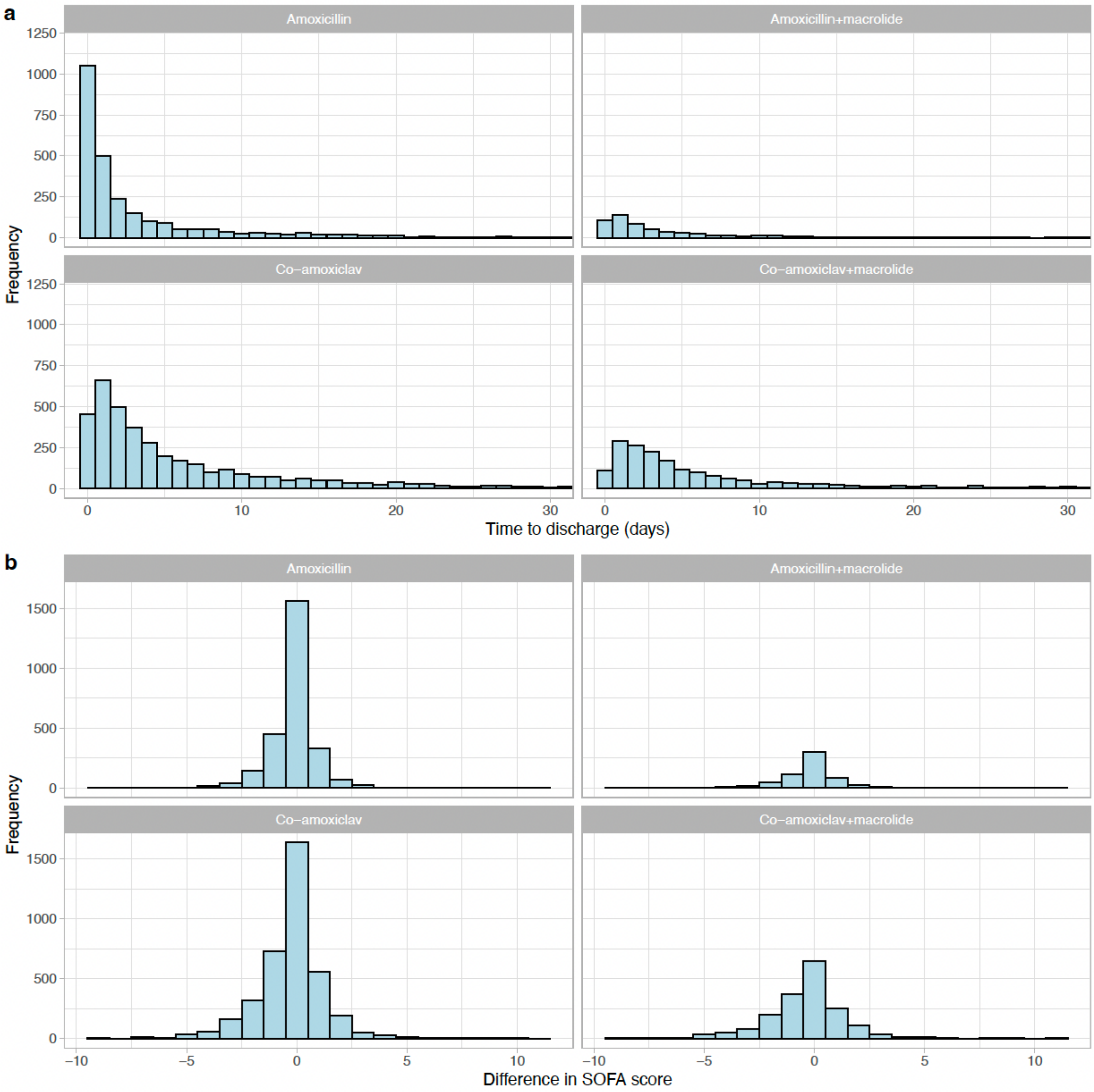

Supplementary Figure 3. Time to hospital discharge in days (a) and difference in Sequential Organ Failure Assessment (SOFA) score at 48h (b) by initial antibiotic treatment.

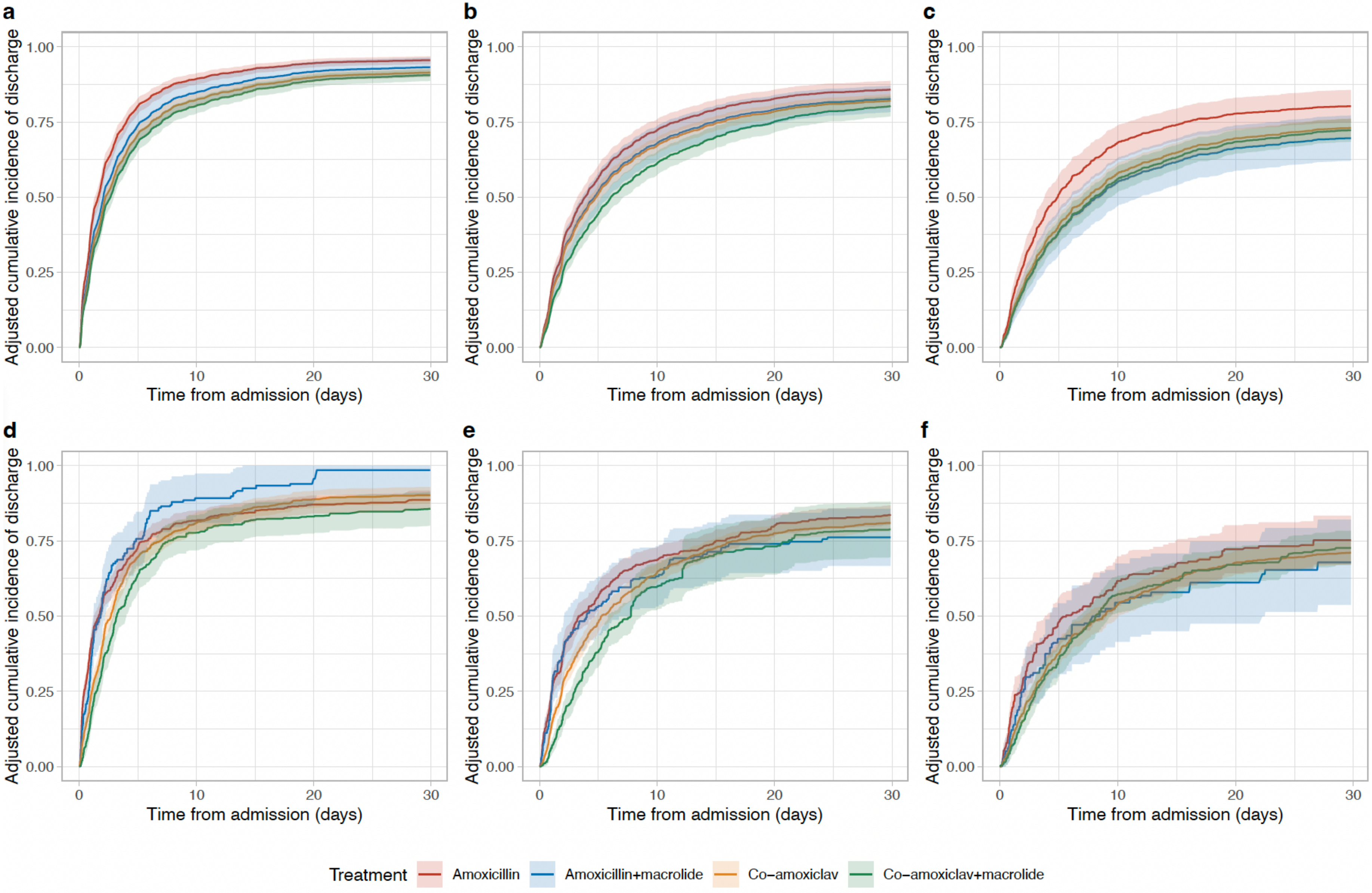
a
b
c
d
e
f
Adjusted cumulative incidence of discharge
1.00
0.75
0.50
0.25
0.00
0
10
20
30
Time from admission (days)
Treatment
Amoxicillin
Amoxicillin+macrolide
Co−amoxiclav
Co−amoxiclav+macrolide

Supplementary Figure 4. Adjusted cumulative incidence (95% confidence intervals) of hospital discharge before death by initial antibiotic treatment in subgroup analyses stratified by baseline pneumonia severity. Time to discharge was censored at 30 days following admission. Severity was determined by CURB-65 score: severe pneumonia (score 3-5), moderate pneumonia (score 2), and mild pneumonia (score 0-1). **Panel a and d** show mild pneumonia, **panel b and e** show moderate pneumonia, **panel c and f** show severe pneumonia. **Panel a, b, c** show estimates using multivariable regression without weighting, while **panel d, e, f** show estimates using inverse probability of treatment weighting (IPTW).
